# ATP Signaling Controlling Dyskinesia Through P2X7 Receptors

**DOI:** 10.3389/fnmol.2020.00111

**Published:** 2020-08-07

**Authors:** Analu A. Fonteles, Julliana C. S. Neves, Ana Paula F. Menezes, Juliana F. Pereira, Ana Thais A. Silva, Rodrigo A. Cunha, Geanne M. Andrade

**Affiliations:** ^1^Post-Graduate Program in Pharmacology, Department of Physiology and Pharmacology, Federal University of Ceará, Fortaleza, Brazil; ^2^Post-Graduate Program in Medical Sciences, Department of Medicine, Faculty of Medicine, Center for Research and Drug Development (NPDM), Federal University of Ceará, Fortaleza, Brazil; ^3^CNC-Center for Neuroscience and Cell Biology, Coimbra, Portugal; ^4^Faculty of Medicine, University of Coimbra, Coimbra, Portugal

**Keywords:** Parkinson’s disease, dyskinesia, P2X7 receptor, neuroinflammation, dopamine D1 receptor, striatum, microglia, astrocyte

## Abstract

Dopamine replacement therapy with L-3,4-dihydroxyphenylalanine (L-DOPA) is the only temporary therapy for Parkinson’s disease (PD), but it triggers dyskinesia over time. Since dyskinesia is associated with increased neuronal firing that bolsters purinergic signaling, we now tested whether the selective and blood-brain barrier-permeable P2X7 receptor antagonist Brilliant Blue-G (BBG, 22.5–45 mg/kg ip) attenuated behavioral, neurochemical and biochemical alterations in rats turned hemiparkinsonian upon unilateral striatal injection of 6-hydroxydopamine (6-OHDA) and treated daily with L-DOPA (30 mg/kg by gavage) for 22 days. The blockade of P2X7 receptors decreased L-DOPA-induced dyskinesia and motor incoordination in hemiparkinsonian rats. In parallel, BBG treatment rebalanced the altered dopamine D1 and D2 receptor density and signaling as well as some neuroinflammation-associated parameters in the striatum and substantia nigra. These findings herald a hitherto unrecognized role for purinergic signaling in the etiopathology of dyskinesia and prompt P2X7 receptor antagonists as novel candidate anti-dyskinesia drugs.

## Highlights

- A P2X7 receptor (P2X7R) antagonist prevents L-DOPA-induced dyskinesia (LID)
- P2X7R blockade prevents LID-induced striatal D1 receptor upregulation
- P2X7R blockade dampens LID-induced DARPP-32 overactivation in striatum and nigra
- P2X7R blockade lowers LID-induced microgliosis and inflammation in striatum and nigra

## Introduction

Parkinson’s disease (PD) is a neurodegenerative disease mainly characterized by bradykinesia resulting from dopamine deficits in the striatum and loss of dopamine neurons in the *substantia nigra*. Dopamine replacement therapy with L-3,4-dihydroxyphenylalanine (L-DOPA) temporarily alleviates PD motor symptoms, although it is accompanied by evolving adverse side effects, namely the development of abnormal involuntary movements known as L-DOPA-induced dyskinesia (LID), occurring in up to 80% of patients within 5 years of treatment (Bastide et al., 2015). LID involves maladaptive changes of dopamine receptors typified by upregulation of dopamine D1 receptor signaling in the striatum (Heumann et al., 2014), altered patterns of synaptic plasticity at corticostriatal synapses (Wang and Zhang, 2016), together with glia deregulation and a heightened profile of neuroinflammation in nigra and striatum (Carta et al., 2017). Thus, dyskinesia resulting from L-DOPA treatment in Parkinsonian rodents triggers an increased firing rate and synaptic potentiation in D1 receptor-containing medium spiny neurons, accompanied by a decreased excitability of D2 receptor-containing medium spiny neurons in the striatum (e.g., Thiele et al., 2014; Suarez et al., 2016), coupled with disrupted excitability and synchronization in both intra- and inter-basal ganglia nuclei and the cerebral cortex. Indeed, the average firing rate of striatal medium spiny neurons increases as axial dyskinesia develops with increased delta power in the striatum (Alberico et al., 2017), hyperexcitability in the subthalamic nucleus (Aristieta et al., 2016), enhanced firing rate in the substantia nigra pars reticulata (Meissner et al., 2006) and overactivation of premotor and motor cortical areas (reviewed in Donzuso et al., 2020).

Notably, increased neuronal firing (Wieraszko et al., 1989; Cunha et al., 1996), as well as increased inflammation (Gourine et al., 2007) and microglia-mediated neuroinflammation (George et al., 2015) are associated with an augmented release of ATP, which acts as a danger signal in the brain (Rodrigues et al., 2015). Accordingly, in different animal models of PD, the blockade of ATP-activated P2X7 receptor (P2X7R) attenuates motor dysfunction (Marcellino et al., 2010; Carmo et al., 2014; Ferrazoli et al., 2017; Wang et al., 2017). This likely involves an ability of P2X7R blockade to attenuate neuroinflammation (reviewed in Volonté et al., 2012; Bartlett et al., 2014; Jimenez-Mateos et al., 2019) and neurodegeneration (e.g., Zhang et al., 2005; Arbeloa et al., 2012; Nishida et al., 2012; Gandelman et al., 2013), in particular, dopaminergic dysfunction (Jun et al., 2007; Carmo et al., 2014; Kumar et al., 2017). The combined evidence that P2X7R controls neuroinflammation and abnormal dopaminergic signaling, and the implication of these two mechanisms to generate LID led us to post the new hypothesis that P2X7R may be involved in LID pathophysiology. The relevance of this aim is best heralded by the current inexistence of therapeutic strategies to manage LID. Thus, based on the previous observations that the blood-brain barrier permeable and selective P2X7R antagonist, brilliant blue G (BBG), attenuated motor incoordination in a model of Huntington’s disease (Díaz-Hernández et al., 2009), we now evaluated if this P2X7R antagonist alleviates LID in a rat model.

## Materials and Methods

### Animals and Drug Treatments

Male *Wistar* rats weighing 250–300 g (*n* = 64) were obtained from the Animal House of the Physiology and Pharmacology Department of Federal University of Ceará. All procedures followed the ARRIVE guidelines (McGrath et al., 2010) and were approved by the ethics committee of the Federal University of Ceará (107/14). Animals were housed four per cage under a 12 h light/dark cycle and behavioral tests were performed between 9:00 AM and 5:00 PM.

**Figure 1:**
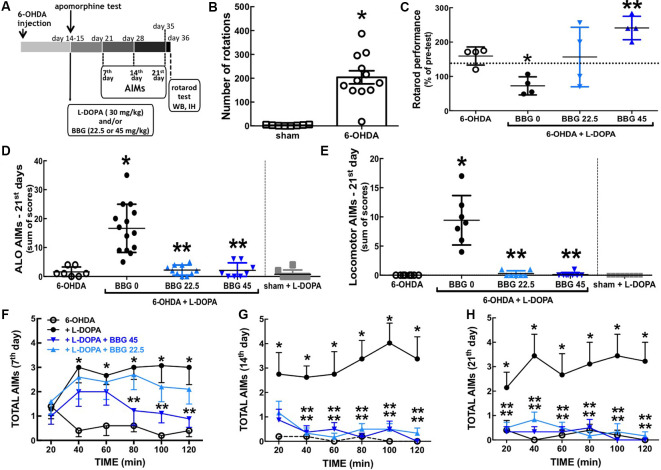
The P2X7R antagonist, brilliant blue G (BBG), prevented L-DOPA-induced dyskinesia and motor incoordination in 6-OHDA-induced hemiparkinsonian rats. **(A)** Schematic overview of the experimental schedule. **(B)** Fourteen days after intra-striatal unilateral injections of 6-OHDA (18 μg/3 μl), rats displayed a sharp increase of apomorphine (1 mg/kg, IP)-induced contralateral rotations counted for 60 min; values are mean ± SEM; *p < 0.05 vs. sham-operated, analysis of variance (ANOVA) followed by a Tukey test (*n* = 8). **(C)** BBG (22.5 or 45 mg/kg, IP) prevents the decreased performance in the rotarod with constant speed (20 rpm) after 22 days of L-DOPA-treatment of hemiparkinsonian rats; the test is specifically designed to evaluate the impact of L-DOPA since the performance is scored as the relative time in the rod 1 h after L-DOPA (30 mg/kg, vo) or saline administration compared to a pre-test done 1 h before L-DOPA (or saline). With all animals trained daily during the 3 days preceding the test in an accelerated version of the rotarod (16–38 rpm during 300 s); the dashed line is the median performance of sham-operated rats; values are medians and spreading intervals; *p < 0.05 vs. 6-OHDA and **p < 0.05 vs. 6-OHDA + L-DOPA, Mann–Whitney test (*n* = 4). **(D,E)** BBG (22.5 or 45 mg/kg, ip) prevents the development of both axial, limb and orolingual (ALO) abnormal involuntary movements (AIMs; **D**) and locomotor AIMs **(E)** after 21 days of daily treatment with L-DOPA of 6-OHDA-induced hemiparkinsonian rats; ALO AIMs (rating as a contralateral side of the lesion; limb AIMs: repetitive, rhythmic jerky movements or dystonic posturing of the forelimb on the contralateral side of the lesion; orolingual AIMs: tongue protusion without the presence of food or other objects) and locomotor AIMs (rating locomotion to the contralateral side of the lesion) were scored as (0), absent; (1), present for less than half of the observation time; (2), present for more than half of the observation time; (3), continuous but interrupted by strong sensory stimuli; (4), continuous, not interrupted by strong sensory stimuli; AIMs were scored as the sum of AIMs observed during 2 min every 20 min, starting 20 min after L-DOPA administration for a total time of 2 h. **(F–H)** Time courses of onset and evolution of total AIMs (sum of locomotor AIMs and ALO AIMs) after the administration of the last L-DOPA dose at 7 days **(F)**, 14 days **(G)** and 21 days of treatment **(H)**. *p < 0.05 vs. 6-OHDA and **p < 0.05 vs. 6-OHDA + L-DOPA using a Kruskal–Wallis test followed by a Dunn’s test (*n* = 8).

The analysis of rotational behavior was carried out using 16 animals, eight for each group. Another pool of 48 animals was used to assess the pattern of AIMs and was divided into six groups: (i) sham-operated rats receiving ascorbate (0.01% in saline; Sigma) intra-striatally; (ii) BBG-treated sham-operated rats (sham+BBG45) receiving ascorbate (0.01% in saline; Sigma) intra-striatally and BBG (45 mg/kg, ip; Sigma); (iii) hemiparkinsonian (6-OHDA) rats receiving an intra-striatal unilateral injection of 6-hydroxydopamine (6-OHDA, 18 μg/3 μl; Sigma), as previously described (Carmo et al., 2014); (iv) dyskinetic (6-OHDA+L-DOPA) rats receiving 6-OHDA as above and L-DOPA (30 mg/kg; Hoffman-Laroche) by gavage daily for 22 days; (v) BBG-treated (6-OHDA+L-DOPA+BBG 22.5) rats receiving 6-OHDA, L-DOPA and BBG 22.5 mg/kg ip; and (vi) BBG-treated (6-OHDA+L-DOPA+BBG 45) rats receiving 6-OHDA, L-DOPA and BBG 45 mg/kg ip for 22 days. After completing an AIMs analysis, four animals of each of these six groups (24 in total) were subjected to the rotarod test, and their brains were dissected and used for western blot analysis; additionally, four animals of each of the six groups (24 in total) were used for immunohistochemical analysis, as depicted in Figure 1. Note that the animals subjected to L-DOPA and/or BBG treatment were previously subjected to apomorphine tests because only animals with recognized striatal lesions caused by 6-OHDA could be used in the following protocols (all except three animals reached this criterium).

The dose range of BBG used in the present study has previously been shown to yield a brain concentration of 200–220 nM (Díaz-Hernández et al., 2012), which is within the effective and selective range of BBG towards central P2X7R (Donnelly-Roberts and Jarvis, 2007). Accordingly, this dose range of BBG has previously been shown to afford robust neuroprotection in different animal models of brain disease (Ryu and McLarnon, 2008; Díaz-Hernández et al., 2009; Arbeloa et al., 2012; Kimbler et al., 2012; Carmo et al., 2014).

### Behavioral Analysis

Rotational behavior was tested with apomorphine (1.0 mg/kg) 14 days after 6-OHDA injection, as previously described (Carmo et al., 2014). Abnormal involuntary movements (AIMs) were quantified as ALO AIMs (summing axial AIMs, limb AIMs, and orolingual AIMs) that are distinguished from locomotor AIMs, and were scored (Lundblad et al., 2002) for 2 min every 20 min during 2 h periods on the 21st, 28th and 36th days after 6-OHDA injection, i.e., 1–3 weeks after the apomorphine test, and during the treatment with L-DOPA without or with BBG. ALO AIMs (rating axial AIMs: contralateral torsions of the neck and body; limb AIMs: repetitive, rhythmic jerky movements or dystonic posturing of the forelimb on the contralateral side of the lesion; orolingual AIMs: tongue protrusion without the presence of food or other objects) and locomotor AIMs (rating locomotion to the contralateral side of the lesion) were scored as: (0), absent; (1), present for less than half of the observation time; (2), present for more than half of the observation time; (3), continuous but interrupted by strong sensory stimuli; and (4), continuous, not interrupted by strong sensory stimuli.

Motor coordination and balance were tested using an accelerated rotarod (Gonçalves et al., 2017), on the 37th day after 6-OHDA injection. Animals were pre-trained two sections a day, 3 days before the test in an accelerated version of the rotarod (16–38 rpm during 300 s). Twenty days after L-DOPA-treatment, each animal was tested 1 and 2 h after L-DOPA (30 mg/kg, v.o.) or saline administration with constant speed (20 rpm) and two consecutive latencies were recorded for each animal up to a maximum of 300 s. Performance was scored as the percentage of the average latency time spent on the rod, 1 and 2 h after L-DOPA, in relation to the average latency time spent in the pre-test performed 30 min before L-DOPA administration. Thus, this protocol is specifically designed to estimate L-DOPA-induced motor incoordination in Parkinsonian models (Padovan-Neto et al., 2009) rather than the impact of 6-OHDA on motor incoordination.

### Histochemical Analysis

After behavioral analysis, four rats from each group were processed as previously described (see Carmo et al., 2014) for tyrosine hydroxylase (TH; 1:800, Sigma) immunohistochemistry and FluoroJade-C staining to evaluate neuronal damage, as well as astrogliosis (GFAP immunoreactivity; 1:1,000, Sigma), microgliosis (CD11b immunoreactivity; 1:200, Serotec), and immunoreactivity for cyclooxygenase-2 (COX-2; 1:200, Santa Cruz Biotechnology, Dallas, TX, USA) and dopamine transporters (DAT; 1:500, Santa Cruz Biotechnology, Dallas, TX, USA). Coronal brain sections (50 μm thick) were collected in three series with an inter-series interval of 300 μm from the beginning to the end of the striatum (approximately 0.96 mm to −0.92 mm from bregma; Paxinos and Watson, 2005) and from the beginning to the end of the substantia nigra (approximately −4.44 mm to −6.19 mm from bregma; Paxinos and Watson, 2005). The stainings were carried out in triplicate using one-in-six free-floating sections. The stained sections were visualized using confocal microscopy (LSM510, Zeiss) and two photographs were collected per section, with care to maintain the conditions of acquisition as constant and without any digital manipulation of the acquired data. After a freehand selection of a 50 × 50 μm region of interest in the striata or nigra (ipsilateral and contralateral), we measured total fluorescence (for TH, GFAP, CD11b or FluoroJade-C) or counted the number of positively-stained elements (for COX-2) using a densitometric analysis based on the ImageJ software (Carmo et al., 2014).

### Western Blot Analysis

After behavioral analysis, four rats from each group were processed for Western blot analysis (Shen et al., 2013), to measure dopamine D1 receptors (1:500, Abcam), dopamine D2 receptors (1:800, Abcam) or interleukin-1β (IL1β, 1:500, Santa Cruz Biotechnology, Dallas, TX, USA), followed by re-probing with α-tubulin (1:500, Santa Cruz Biotechnology, Dallas, TX, USA), and phospho-threonine-34 DARPP-32 (p-DARPP-32, 1:1,500, Santa Cruz Biotechnology, Dallas, TX, USA) followed by total DARPP-32 (1:1,500, Cell Signaling) re-probing (Shen et al., 2013).

### Statistical Analysis

All statistical comparisons were performed using Graphpad Prism 6.0 software, with a significance level of 95%. Rotational test performance, D1 and D2 receptors, DARPP-32 and IL-1β Western-blot analysis, and COX-2 immunohistochemical analysis are represented as mean ± SEM and statistical differences were estimated using one-way analysis of variance (ANOVA), followed by a Tukey’s test. The analysis of AIMs and of rotarod performance, as well as TH, DAT, GFAP and CD11b immunohistochemical results are presented as median (interquartile range) and were analyzed with Kruskal-Wallis tests followed by Mann–Whitney U-tests to compare pairs of groups.

## Results

Intra-striatal 6-OHDA injection triggered dopamine depletion and super-sensitization, as heralded by the sharp increase of apomorphine-induced contralateral rotations 14 days after 6-OHDA (*F*_(2,25)_ = 10.99, *p* < 0.0001; Figure 1B). Chronic L-DOPA (30 mg/kg) treatment of 6-OHDA-lesioned rats significantly increased axial, limb and orolingual AIMs (*F*_(4,39)_ = 20.61, *p* = 0.0005; Figure 1D) as well as locomotor AIMs (*F*_(5,38)_ = 17.63, *p* = 0.0020; Figure 1E). No AIMs were observed in sham-operated animals after receiving chronic L-DOPA treatment or vehicle (Figures 1D,E).

### Blockade of P2X7R Attenuated L-DOPA-Induced Dyskinesia

Chronic treatment with the selective P2X7R antagonist, BBG (45 mg/kg) diminished AIMs’ scores 21 days after L-DOPA treatment of hemiparkinsonian rats (Figures 1D,E; *F*_(4,14)_ = 19.51; *p* = 0.0006 and *F*_(4,17)_ = 19.56; *p* = 0.0004, respectively), as also occurred 7–14 days after L-DOPA treatment (Figures 1F,G). BBG (45 mg/kg) decreased AIMs’ scores within 80 min after L-DOPA injection on day 7, within 40 min on day 14, and within 20 min on day 21 (Figures 1F–H). A lower dose of BBG (22.5 mg/kg) also attenuated AIMs’ scores (Figures 1D,E; *F*_(4,14)_ = 18.02; *p* = 0.0007 and *F*_(4,12)_ = 17.07; *p* = 0.0048, respectively). We have previously reported that the tested doses of BBG are devoid of motor effects in control rats (Carmo et al., 2014).

### Blockade of P2X7R Attenuated LID-Induced Motor Incoordination

Chronic L-DOPA treatment decreased motor coordination of hemiparkinsonian rats on the rota-rod test (*F*_(4,16)_ = 5.249; *p* = 0.0068), which was prevented by BBG (45 mg/kg; *F*_(4,16)_ = 6.477; *p* = 0.025; Figure 1C). No alterations were observed in sham-operated animals chronically treated with L-DOPA or vehicle (not shown) and the impact of BBG on 6-OHDA-treated rats was previously described (Carmo et al., 2014).

### Blockade of P2X7R Dampened Dopamine Aberrant Signaling Associated With LID

**Figure 2:**
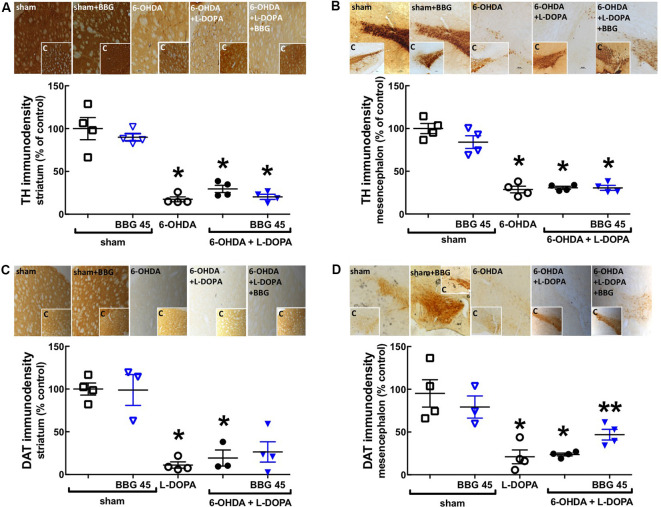
The P2X7R antagonist, brilliant blue G (BBG), applied after L-DOPA-induced dyskinesia, did not recover the 6-OHDA-induced dopaminergic lesion in the striatum and nigra. Rats were either sham-operated or challenged with 6-OHDA (unilateral striatal injection of 18 μg/3 μl) and later treated either with saline (PARK) or L-DOPA (30 mg/kg, v.o. daily) or L-DOPA plus BBG (45 mg/kg, IP daily, 30 min before L-DOPA), before being sacrificed for preparation of coronal brain sections (30 μm) to be immunohistochemically stained with the dopaminergic markers tyrosine hydroxylase (TH) or dopamine transporter (DAT). The treatment during 21 days of L-DOPA and/or BBG does not affect the 6-OHDA-induced dopaminergic lesion in the ipsilateral striatum **(A,C)** and in the contralateral substantia nigra **(B,D)**, assessed by immunohistochemical analysis of either TH **(A,B)** or dopamine transporters (DAT; **C,D**); the photographs in each panel are representative stainings ordered as the columns and the inserts are photographs from the other hemisphere (as internal controls); the values in the bar graphs are mean ± SEM of four rats per group; *p < 0.05 vs. sham-operated using a Mann–Whitney test. **p < 0.05 vs. 6-OHDA + L-DOPA using a Mann-Whitney test.

Rats with 6-OHDA lesions exhibited a greater than 70% reduction (*p* = 0.0286) of the immunodensity of the dopamine markers tyrosine hydroxylase (TH; Figures 2A,B) and of dopamine transporters (DAT; *p* = 0.0009; Figures 2C,D) only in the ipsilateral striatum (Figures 2A,C) and contralateral substantia nigra (Figures 2B,D; *p* = 0.0286 and *p* = 0.0286, respectively) compared to the sham-operated group; this was not affected by chronic treatment with L-DOPA and/or BBG (Figure 2). As expected when beginning BBG treatment after establishing dopamine neuronal lesions, there was no change of FluoroJade-C staining (degenerating neurons) in the striatum or nigra between the different groups (data not shown).

**Figure 3:**
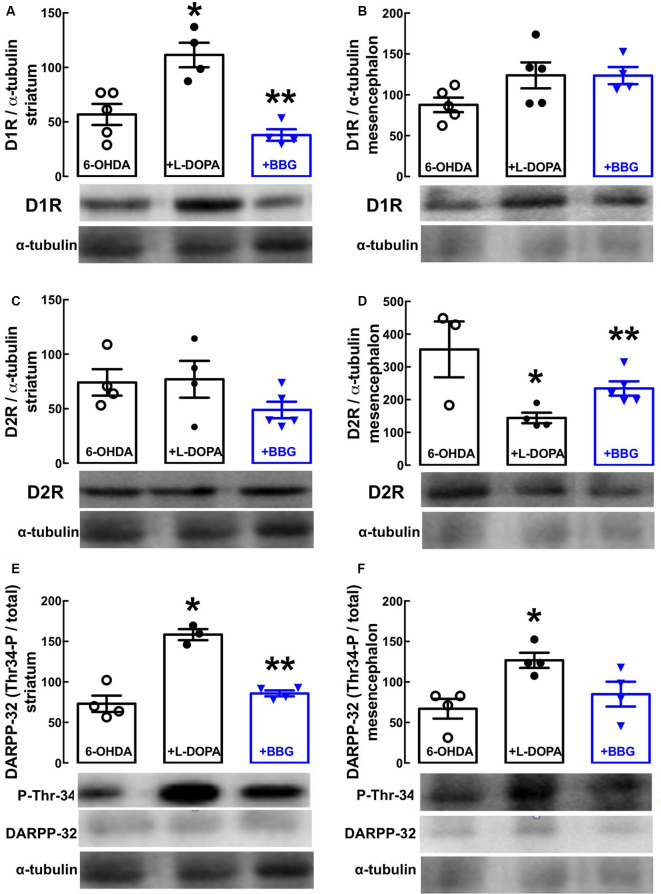
The P2X7R antagonist, brilliant blue G (BBG), prevents the alterations of the dopaminergic signaling associated with the altered density of dopamine D1 and D2 receptors in the striatum and substantia nigra of hemiparkinsonian rats suffering from L-DOPA-induced dyskinesia. Rats were challenged with 6-OHDA (unilateral striatal injection of 18 μg/3 μl) and either saline (6-OHDA) or L-DOPA (30 mg/kg, v.o. daily, +L-DOPA) or L-DOPA plus BBG (45 mg/kg, IP daily, 30 min before L-DOPA; +BBG), and were sacrificed for preparation of extracts from the striatum or substantia nigra for Western blot analysis. **(A,B)** D1 receptor immunodensity in the striatum **(A)** and nigra **(B)**. **(C,D)** D2 receptor immunodensity in the striatum **(C)** and nigra **(D)**. **(E,F)** Threonine-34-phosphorylated DARPP-32 immunodensity in the striatum **(E)** and nigra **(F)**. All data are mean ± SEM of four rats per group; *p < 0.05 vs. sham-operated, **p < 0.05 vs. PARK+L-DOPA, using an ANOVA followed by a Tukey’s test.

Western blot analysis revealed a selective increase of dopamine D1 receptor density in the striatum (Figure 3A, *p* = 0.0159) with no alteration in the nigra (Figure 3B, *p* = 0.2119), whereas dopamine D2 receptor density decreased in the nigra (Figure 3D, *p* = 0.0085), but not in the striatum (Figure 3C, *p* = 0.9982) of LID rats (i.e., treated with 6-OHDA and then with L-DOPA) compared to PD rats (i.e., treated only with 6-OHDA). This resulted in an abnormally increased dopaminergic signaling in both the striatum (Figure 3E, *p* = 0.0102) and nigra (Figure 3F, *p* = 0.0106), as testified by an increased ratio of threonine-34-phosphorylated/total DARPP-32 in LID vs. PD rats. Notably, BBG (45 mg/kg) prevented these changes in striatal D1 (Figure 3A, *p* = 0.0304) and nigra D2 receptors (Figure 3D, *p* = 0.0159), normalizing dopamine signaling through DARPP-32 in both the striatum (*p* = 0.0297) and nigra (*p* = 0.0596) of LID rats (Figures 3E,F).

### Blockade of P2X7R Prevented Some Glia-Associated Neuroinflammation Markers Upon LID

**Figure 4:**
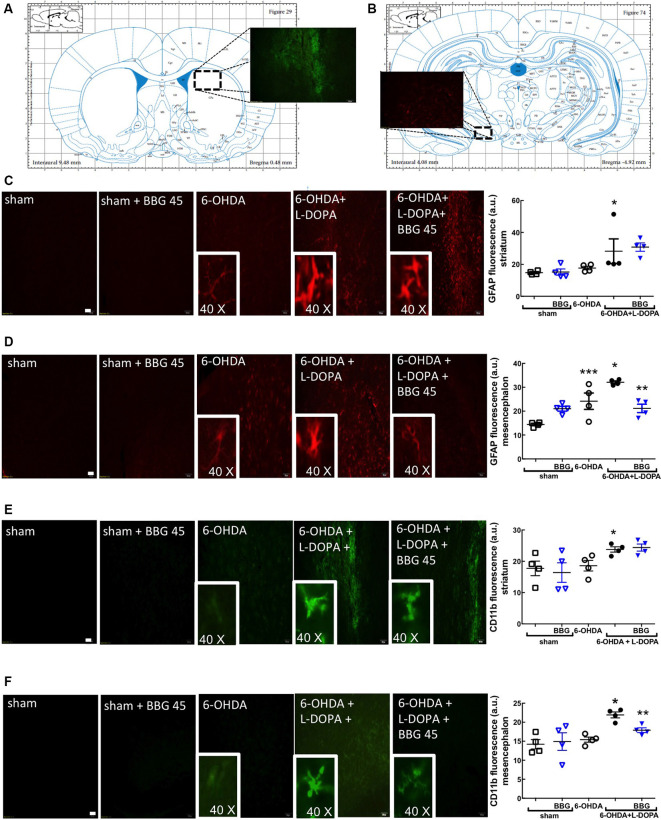
The P2X7R antagonist, brilliant blue G (BBG), prevented astrocytic and microglial alterations in the striatum **(A)** and substantia nigra **(B)** of hemiparkinsonian rats suffering from L-DOPA-induced dyskinesia. Rats were either sham-operated or challenged with 6-OHDA (unilateral striatal injection of 18 μg/3 μl) and later treated either with saline or L-DOPA (30 mg/kg, v.o. daily) or L-DOPA plus BBG (45 mg/kg, IP daily, 30 min before L-DOPA), before being sacrificed for preparation of either coronal brain sections (50 μm thick) or extracts from the striatum or substantia nigra for Western blot analysis. **(A)** Representation from the rat brain Atlas of Paxinos and Watson (2005) at 0.48 mm from bregma, illustrating the site of a collection of striatal sections (50 μm thick) in three series of six sections around the three 6-OHDA injection sites and **(B)** representation at −4.92 mm from bregma, illustrating the site of a collection of nigral sections (50 μm thick) in three series of six sections; the insert photograph shows an example of a striatal **(A)** and nigral regions **(B)** stained for CD11b from a 6-OHDA + L-DOPA treated rat. **(C,D)** Representative photographs (ordered as indicated in the bar graph showing the average staining values) of the immunohistochemical detection of the astrocyte marker, GFAP in the striatum **(C)** and nigra **(D)**, where the inserts are the amplification of individually stained elements; bar values are medians and spreading intervals; *p < 0.05 vs. sham-operated, **p < 0.05 vs. 6-OHDA and ***p < 0.05 vs. 6-OHDA + L-DOPA, Mann–Whitney test (*n* = 4). **(E,F)** Representative photographs (ordered as indicated in the bar graph showing the average staining values) of the immunohistochemical detection of the microglia marker, CD11b in the striatum **(E)** and nigra **(F)**, where the inserts are the amplification of individually stained elements; bar values are medians and spreading intervals; *p < 0.05 vs. sham-operated, **p < 0.05 vs. 6-OHDA and ***p < 0.05 vs. 6-OHDA + L-DOPA, Mann–Whitney test (*n* = 4). Scale bar: 100 μm.

**Figure 5:**
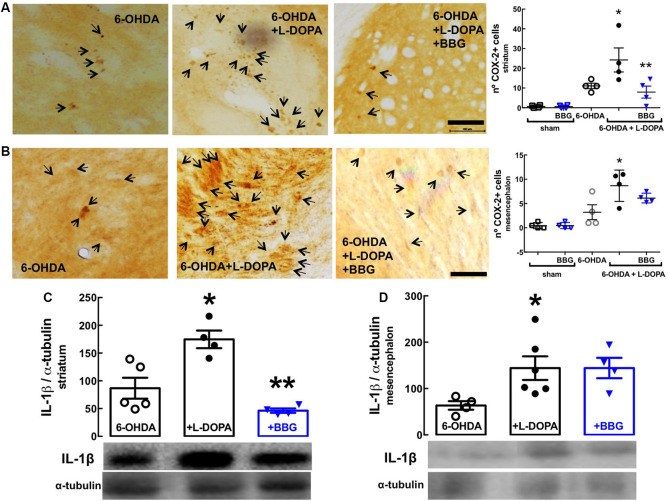
The P2X7R antagonist, brilliant blue G (BBG), prevented alterations associated with neuroinflammation in the striatum and substantia nigra of hemiparkinsonian rats suffering from L-DOPA-induced dyskinesia. Rats were either sham-operated or challenged with 6-OHDA (unilateral striatal injection of 18 μg/3 μl) and later treated either with saline or L-DOPA (30 mg/kg, v.o. daily) or L-DOPA plus BBG (45 mg/kg, IP daily, 30 min before L-DOPA), before being sacrificed for preparation of either coronal brain sections (50 μm thick) or extracts from the striatum or substantia nigra for Western blot analysis. **(A,B)** The average immunohistochemical density of cyclooxygenase-2 (COX-2) in the striatum **(A)** and nigra **(B)**; bar values are mean ± SEM of four rats per group; *p < 0.05 vs. 6-OHDA, **p < 0.05 vs. 6-OHDA + L-DOPA, using an ANOVA followed by a Tukey’s test. **(C,D)** Immunodensity of interleukin-1β determined by Western blot analysis in the striatum **(C)** and nigra **(D)**; bar values are mean ± SEM of four rats per group; *p < 0.05 vs. 6-OHDA, **p < 0.05 vs. 6-OHDA + L-DOPA, using an ANOVA followed by a Tukey’s test. Scale bar: 100 μm.

GFAP (astrocytic marker) and CD11b (microglia/macrophage marker) immunoreactivities were increased in the striatum (Figures 4C,E) and nigra (Figures 4D,F) of 6-OHDA-treated rats and further increased upon LID; this suggests the presence upon LID of astrogliosis and microgliosis, as concluded from the quantification of the total immunoreactivities of GFAP and CD11b (scatter plots in Figure 4), as well as the alteration of the morphology of the elements stained with either GFAP or CD11b (insert photos in Figure 4). These alterations were prevented by BBG (45 mg/kg), more evidently in the nigra than in the striatum (Figure 4). Accordingly, LID increased COX-2 immunoreactivity (Figures 5A,B; Bortolanza et al., 2015b) and interleukin-1β levels in the striatum and nigra (Figures 5C,D), which was prevented by BBG more evidently in the striatum (Figures 5A,C) than in the nigra (Figures 5B,D). Altogether, these findings are indicative of the ability of P2X7R to control neuroinflammation, although BBG displays a different ability to control glial cell markers and neurochemical features of neuroinflammation in the striatum and in the nigra. This might be due to the greater density of microglia in the nigra than elsewhere in the brain (Lawson et al., 1990; Sharaf et al., 2013), the different contribution of different players to mount a neuroinflammatory response in the striatum and in the nigra (Walker et al., 2016) or to the scar caused by the injection of 6-OHDA in the striatum.

## Discussion

Dopamine replacement therapy with L-DOPA is the main temporary therapy for PD but it triggers dyskinesia (LID) over time. We used a commercially available, brain permeable and selective P2X7R antagonist, Brillant Blue G (BBG), to show its ability to prevent LID in a rat model. We confirmed that a unilateral 6-OHDA injection caused a loss of dopamine neurons (TH, DAT staining) in the striatum and nigra, impairing motor responses (apomorphine-induced rotations) consistent with a hemiparkinsonian condition. Chronic L-DOPA administration triggered locomotor, postural, and orofacial dyskinetic movements together with motor incoordination, without altering 6-OHDA-induced dopaminergic degeneration, thus confirming the face validity of the model (Bastide et al., 2015). BBG prevented the development of the critical behavior alterations of LID, namely dyskinesia and motor incoordination. Since the two tested doses of BBG selectively target P2X7R (Donnelly-Roberts and Jarvis, 2007; Díaz-Hernández et al., 2012), having effects similar to other selective P2X7R antagonists and P2X7R knockout in different models of brain diseases (Melani et al., 2006; Díaz-Hernández et al., 2009, 2012; Kimbler et al., 2012; Carmo et al., 2014; Jimenez-Pacheco et al., 2016), the effects of BBG imply the likely involvement of P2X7R.

The pathophysiological changes underlying LID remain poorly understood. One defined neurochemical basis of LID is the imbalance of the nigrostriatal dopaminergic system due to the loss of pulsatile dopamine signaling replaced by a continuous L-DOPA-derived dopamine generation (Heumann et al., 2014). Accordingly, our LID model revealed an increased D1 receptor density, without alteration of the D2 receptor density in the striatum, together with a decreased D2 receptor density without alteration of the D1 receptor density in the nigra. This resulted in abnormal dopaminergic signaling in both structures, as indicated by the increase of DARPP-32 phosphorylation in threonine-34, which is associated with dopaminergic signaling (Svenningsson et al., 2004). BBG prevented all these LID-associated dopaminergic alterations, which might eventually result from direct effects of P2X7R located in dopamine cells (Heine et al., 2007) and striatal dopamine terminals (Carmo et al., 2014). However, although the presence of P2X7R mRNA and protein in the striatum and substantia nigra is established (Amadio et al., 2007; Kaczmarek-Hajek et al., 2018; Crabbé et al., 2019; Gentile et al., 2019) and there is evidence for an increased P2X7R density in the striatum upon decreasing dopaminergic innervation (Ferrazoli et al., 2017; Crabbé et al., 2019), the cellular localization of P2X7R is essentially unknown, and there is no data currently available to ascribe the presence of P2X7R to different neuronal populations (e.g., D1R or D2R) in nigrostriatal pathways.

A concurrent mechanism of action of P2X7R to dampen LID results from the ability of BBG to prevent the alterations of astrocytes and microglia and the putative neuroinflammation suggested by the increased COX-2 and interleukin-1β levels in the striatum and nigra of LID mice. Indeed, neuroinflammation involving microglia and astrocytes critically controls LID (Bortolanza et al., 2015a; Mulas et al., 2016; Carta et al., 2017) and P2X7R are well-established to control neuroinflammation (Rodrigues et al., 2015; Illes et al., 2017), being most abundant in microglia (Melani et al., 2006; Bhattacharya and Biber, 2016; Kaczmarek-Hajek et al., 2018) and astrocytes (Oliveira et al., 2011). We observed that BBG displayed a different efficiency to prevent neuroinflammation-associated neurochemical alterations rather than alterations of glial cells in the striatum and neuroinflammation-associated glial cell alterations rather than neurochemical alterations in the nigra, probably due to the contribution of different players in mounting neuroinflammatory responses in the nigra and striatum (Walker et al., 2016) or as a consequence of the striatal scar caused by 6-OHDA administration. However, some parameters associated with neuroinflammation were attenuated by BBG in the striatum and nigra. Notably, several studies have proposed that the P2X7R-mediated control of neuroinflammation is actually responsible to control neuronal function (Hu et al., 2015; Bernardino et al., 2008) and recent studies identified P2X7R in microglia as paramount to mediate behavioral alterations upon repeated stress (Iwata et al., 2016; Yue et al., 2017). Further studies should exploit this observed robust ability of P2X7R to control dyskinesia as a new window of opportunity to disentangle the relative importance of the control of neuroinflammation and of the maladaptive dopaminergic alterations for the development of LID.

Although we propose that P2X7R might mainly modulate striatal and nigra dopaminergic function and neuroinflammation to control dyskinesia, it is important to note that our results do not allow a definition of the brain area (striatum, nigra or cerebral cortex) where P2X7R might play the more prominent role to control dyskinesia. The time course evaluation of the effects of BBG on dyskinesia also prompted the suggestion that the beneficial effects of BBG seem to increase with time, i.e., they seem more robust after 14 and 21 days of treatment compared to shorter periods of exposure to BBG (7 days). Future studies will be required to explore the apparent time-dependent increase of the efficiency of BBG and potential relation to an increased released of ATP, an upregulation of P2X7R, and/or increased efficiency of P2X7R action in altered cellular networks upon the evolution of a dyskinetic phenotype.

In conclusion, the present study provides the first demonstration for the involvement of the purinergic system in the development of LID and prompts considering P2X7R antagonists as novel candidate anti-dyskinesia drugs.

## Data Availability Statement

The datasets generated for this study will not be made publicly available; videos and lab books can only be scrutinized in loco at the University of Ceará.

## Ethics Statement

The animal study was reviewed and approved by the ethics committee of the Federal University of Ceará (107/14).

## Author Contributions

AF, JN, AM, JP, and AS carried out the experimental manipulations and analyzed the data. AF, GA, and RC planned the experiments. AF, GA, and RC wrote the manuscript.

## Conflict of Interest

RC is a scientific consultant of the Institute for Scientific Information on Coffee (ISIC). The remaining authors declare that the research was conducted in the absence of any commercial or financial relationships that could be construed as a potential conflict of interest.

## Abbreviations

AIMs: abnormal involuntary movements
BBG: Brilliant Blue-G
CD11b: cluster of differentiation 11b
COX-2: cyclooxygenase-2
DARPP-32: dopamine- and cAMP-regulated neuronal phosphoprotein
DAT: dopamine transporters
GFAP: glial fibrillary acidic protein
IL1β: interleukin-1β
L-DOPA: L-3,4-dihydroxyphenylalanine
LID: L-DOPA-induced dyskinesia
6-OHDA: 6-hydroxydopamine
P2X7R: P2X7 receptor
PD: Parkinson’s disease
p-DARPP32: DARPP32 phosphorylated in residue threonine-34
TH: tyrosine hydroxylase.
